# Double Gamers—Can Modified Natural Regulators of Higher Plants Act as Antagonists against Phytopathogens? The Case of Jasmonic Acid Derivatives

**DOI:** 10.3390/ijms21228681

**Published:** 2020-11-17

**Authors:** Nicolò Orsoni, Francesca Degola, Luca Nerva, Franco Bisceglie, Giorgio Spadola, Walter Chitarra, Valeria Terzi, Stefano Delbono, Roberta Ghizzoni, Caterina Morcia, Agnieszka Jamiołkowska, Elżbieta Mielniczuk, Francesco M. Restivo, Giorgio Pelosi

**Affiliations:** 1 Department of Chemistry, Life Sciences and Environmental Sustainability, University of Parma, Parco Area delle Scienze 11/A, 43124 Parma, Italy; nicolo.orsoni@studenti.unipr.it (N.O.); franco.bisceglie@unipr.it (F.B.); giorgio.spadola1@studenti.unipr.it (G.S.); restivo@unipr.it (F.M.R.); giorgio.pelosi@unipr.it (G.P.); 2 Council for Agricultural Research and Economics—Research Centre for Viticulture and Enology CREA-VE, Via XXVIII Aprile 26, 31015 Conegliano (TV), Italy; luca.nerva@crea.gov.it (L.N.); walter.chitarra@crea.gov.it (W.C.); 3 Institute for Sustainable Plant Protection, CNR, Strada delle Cacce 73, 10135 Torino, Italy; 4 Council for Agricultural Research and Economics—Research Centre for Genomics and Bioinformatics CREA-GB, Via San Protaso 302, 29017 Fiorenzuola d’Arda (PC), Italy; valeria.terzi@crea.gov.it (V.T.); stefano.delbono@crea.gov.it (S.D.); roberta.ghizzoni@crea.gov.it (R.G.); caterina.morcia@crea.gov.it (C.M.); 5 Department of Plant Protection, University of Life Sciences in Lublin, Leszczyńskiego 7, 20069 Lublin, Poland; aguto@wp.pl (A.J.); elzbieta.mielniczuk@up.lublin.pl (E.M.)

**Keywords:** jasmonic acid, jasmone derivatives, phytopathogenic fungi, crop protection, thiosemicarbazones, mycotoxins, mycopesticides

## Abstract

As key players in biotic stress response of plants, jasmonic acid (JA) and its derivatives cover a specific and prominent role in pathogens-mediated signaling and hence are promising candidates for a sustainable management of phytopathogenic fungi. Recently, JA directed antimicrobial effects on plant pathogens has been suggested, supporting the theory of oxylipins as double gamers in plant-pathogen interaction. Based on these premises, six derivatives (dihydrojasmone and cis-jasmone, two thiosemicarbazonic derivatives and their corresponding complexes with copper) have been evaluated against 13 fungal species affecting various economically important herbaceous and woody crops, such as cereals, grapes and horticultural crops: *Phaeoacremonium minimum*, *Neofusicoccum parvum*, *Phaeomoniella chlamydospora*, *Fomitiporia mediterranea*, *Fusarium poae*, *F. culmorum*, *F. graminearum*, *F. oxysporum* f. sp. *lactucae,* *F. sporotrichioides*, *Aspergillus flavus*, *Rhizoctonia solani,* *Sclerotinia* spp. and *Verticillium dahliae*. The biological activity of these compounds was assessed in terms of growth inhibition and, for the two mycotoxigenic species *A. flavus* and *F. sporotrichioides*, also in terms of toxin containment. As expected, the inhibitory effect of molecules greatly varied amongst both genera and species; cis-jasmone thiosemicarbazone in particular has shown the wider range of effectiveness. However, our results show that thiosemicarbazones derivatives are more effective than the parent ketones in limiting fungal growth and mycotoxins production, supporting possible applications for the control of pathogenic fungi.

## 1. Introduction

Synthesized from α-linolenic acid originating from chloroplast membranes, jasmonic acid (JA) is regarded as a key player among the molecules that play a role in plant defense, signaling and development; it is known to be involved, together with other phytohormones (e.g., salicylic acid, ethylene, indole-3-acetic acid, abscisic acid, cytokinin and gibberellin), in the host defense response and immune signaling modulation. Above all, it is well known to be involved in the regulation of plant adaptations to biotic (such as pathogen infection and herbivore attack) as well as abiotic stresses [1,2]. Alongside an increasing number of studies showing that JA intervenes in a remarkable number of plant developmental events, including primary root growth, reproductive development and leaf senescence, it has been long-time proven that some plant hormones are also able to affect fungal physiology. In particular, it is worth noting that JA can have an impact on mycelium growth and development, conidiation, spore germination and sexual reproduction, suggesting that these molecules can interfere with specific signal transduction processes in fungi, although the effect strongly depends on the species [3,4,5,6,7]. Some of these molecules were successfully used for the discovery of novel and diverse natural products, being effective in activating silent biosynthetic genes/pathways of fungal secondary metabolism [8]. At the same time, some fungi (and not only plant-interacting ones) have the ability to synthetize plant hormones and their analogs [9,10,11]. With the idea of exploiting the potential of JA derivatives to interfere with fungal development and metabolism, a panel of six JA-derived molecules (two ketones of natural origin, di-hydrojasmone and cis-jasmone, their thiosemicarbazonic derivatives and the corresponding copper complexes) was evaluated against 13 fungal species affecting various economically important herbaceous and woody crops such as cereals, grape and horticultural plants. All these fungi cause relevant yield and quality losses and are characterized by different life cycle, pathogenicity behavior, transmission mode and effects on both plants and derived products. We grouped the investigated fungal species in three classes according to the prevalent host plant: (1) *Neofusicoccum parvum*, *Phaeoacremonium minimum* and *Phaeomoniella chlamydospora*, belonging to the Ascomycota phylum and previously characterized as associated to esca disease in grapevine, one of the main constraints now emerging in viticulture [12]; *Fomitiporia mediterranea*, belonging to the Basidiomycota phylum and known to be the causal agent of white rot in grape wood trunks. (2) *Fusarium culmorum*, *F. graminearum*, *F. sporotrichioides*, *F. poae* and *Aspergillus flavus*, all Ascomycota species affecting grain cereals. *Fusarium* spp. are responsible for *Fusarium* Head Blight (FHB), a disease of great economic concern for both cereal producers and grain processing industry [13]. This disease is most often caused by *F. graminearum* and *F. culmorum* and in recent years also by *F. poae.* The participation of individual species in causing FHB is highly dependent on weather conditions [14,15,16]. *Aspergillus flavus*, as well as *Fusarium* spp., can contaminate the colonized grains with several classes of mycotoxins, secondary metabolites dangerous to human and animal health, severely affecting yield production and lowering the quality and safety of the final products [17,18,19]. (3) *Rhizoctonia solani*, *Sclerotinia* spp., *Fusarium oxysporum* and *Verticillium dahliae*, all soil-borne fungal pathogens, belonging to Basidiomycota and Ascomycota divisions, etiological agents of several diseases in a wide range of plants worldwide. Even though different in their life cycle, they share the responsibility for affecting agricultural yield losses, especially in the horticultural crops cultivated in temperate regions [20].

## 2. Results

### 2.1. Chemistry

**Scheme 1:**
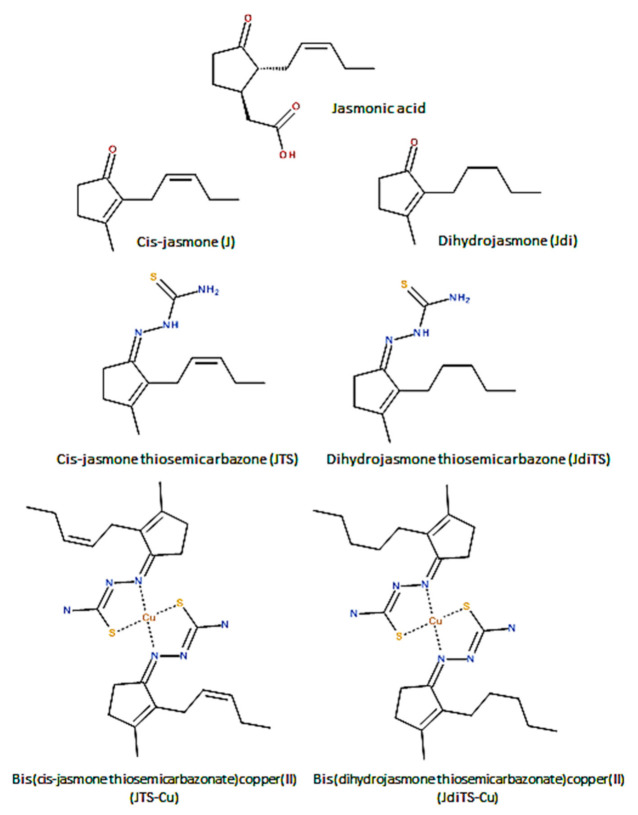
Schematic representation of the structures of Jasmonic acid (JA) derivatives (J, Jdi), their thiosemicarbazones (JTS, JdiTS), and their copper complexes (JTS-Cu, JdiTS-Cu).

**Figure 1:**
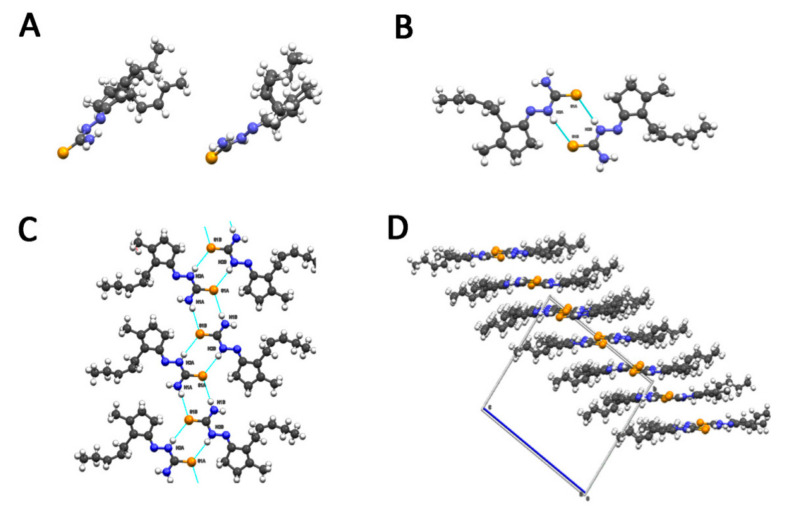
(**A**) Overlapping of molecules A/B (left) and A/C (right). (**B**) Hydrogen bonding scheme between molecule A and B. (**C**) Hydrogen bond scheme along the ribbons formed by molecules A/B. (**D**) packing of the ribbons. Sulfur is represented in yellow, nitrogen in blue and carbon in gray.

As stated in the introduction, a panel of six molecules was used to test their effect on the selected fungal species. The compounds are two ketones of natural origin, cis-jasmone and dihydrojasmone, their thiosemicarbazone derivatives and the corresponding copper complexes (Scheme 1). The choice for a derivatization has fallen on thiosemicarbazones because they are a versatile class of compounds that possess biological properties such as antibacterial, antivirus, antiamoebic and antitumor in human pharmacology [21]. It is also well known that inorganic substances, like copper salts, have long been used for their capacity of inhibiting the development of molds and bacteria and can have effect on the growth of fungi and on aflatoxin production [22]. Moreover, thiosemicarbazone metal complexes and those of copper(II) in particular, are known to produce interesting biological effects showing that the coordination compounds own effective properties at lower concentrations with respect to the parent organic molecules and these properties are often related to reactive oxygen species (ROS) production or other pathways involving the metal ion [23]. All the compounds used in this study were characterized by standard methods and for one of them (JTS) the structure by X-ray diffraction (XRD) was also obtained (Figure 1). The structure is particularly interesting because it reveals that the hydrophobic lateral chain of the jasmone moiety molecule possesses a certain degree of freedom. The asymmetric unit of JTS (CCDC 2019086) consists of three independent molecules—two of them (A and B) form a dimeric structure while the third molecule (C) forms a dimer with a symmetry related molecule C. Molecule A in particular differs from molecules B and C by the orientation of the hydrophobic chain. Both dimers (A:B and C:C’) present a double intermolecular hydrogen bond between the N2 of one molecule and the S1 of the other (Table 1). Bond lengths in the thiosemicarbazone moiety of the three molecules were calculated and reported as average in Table 2.

Sulfur S1 of the thiosemicarbazone is involved in a second intermolecular hydrogen bond with N4-H of a third molecule (Figure 1C). This bond is responsible for the formation of a second type of dimer-like structures which form ribbons developing along the *a* axis. As shown in Figure 1D, the packing of the crystal can be described as due to the stacking of planes formed alternatively by ribbons of heterodimers (A and B) and homodimers (C and C’). These layers are kept together by hydrophobic and π-π stacking interactions of the thiosemicarbazone moiety).

### 2.2. Scavenging Potential Determination of Compounds

**Figure 2:**
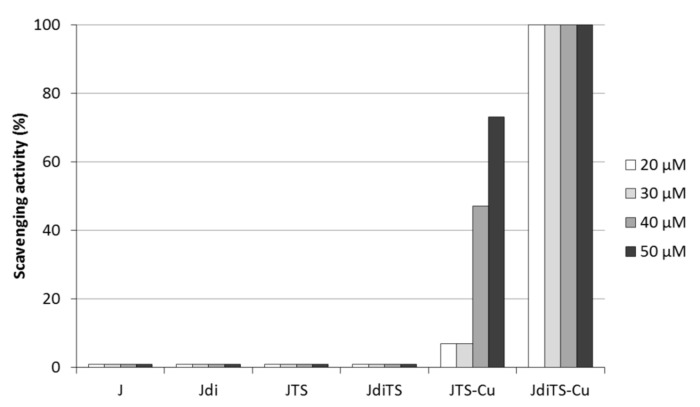
Scavenging assay (2,2-diphenyl-1-picrylhydrazyl radical (DPPH)). The in vitro antioxidant ability of compounds is expressed as percentage of inhibition with respect to ascorbic acid 30 mM scavenging activity (100%).

The scavenging potential of thiosemicarbazones and of their metal complexes was assessed against the 2,2-diphenyl-1-picrylhydrazyl radical (DPPH), by measuring the bleaching of a purple colored methanol solution of the stable DPPH radical [24]. The DPPH radical scavenging ability of JdiTS-Cu traced those of 0.3 mM ascorbic acid (100%) at each concentration tested, while JTS-Cu showed a dose-dependent potential that reached 45% of DPPH radical inhibition at 40 µM concentration and overcame 70% at 50 µM (Figure 2). On the contrary, both aldehydes (J and Jdi) and their thiosemicarbazones derivatives did not show any appreciable scavenging activity at any tested concentration. These observations suggested that the complexation with copper significantly increases the otherwise slight proton-donating ability of ligands, providing them with more interesting potential as free radical inhibitors - or scavengers, acting possibly as primary antioxidants.

### 2.3. Antifungal Activity on Grapevine Esca-Associated Pathogens

**Figure 3:**
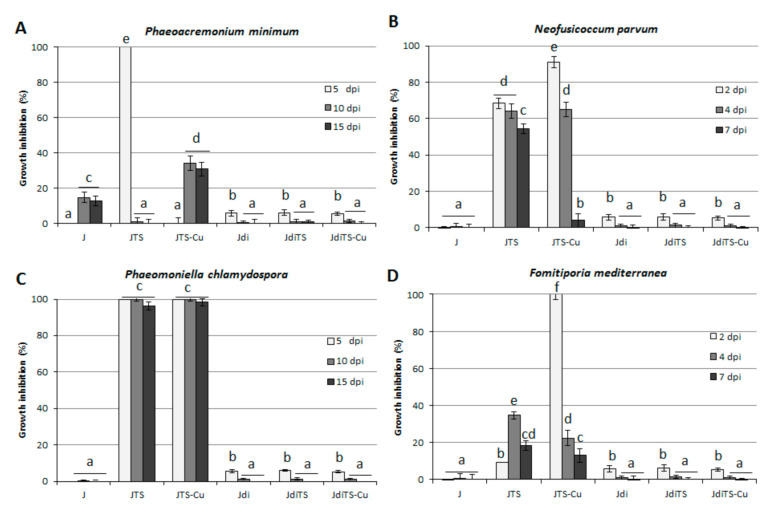
Effect of compounds on the growth of esca disease-linked species (**A**) *P. minimum*, (**B**) *N. parvum*, (**C**) *P. chlamydospora* and (**D**) *F. mediterranea*. Culture medium was amended with molecules at 25 μM concentration; values are reported as inhibition percentage as compared to the control (Dimethyl sulfoxide, DMSO, 0.25% *v/v* amended cultures) ± S.D. Different letters indicate statistically significant differences (*p* < 0.05).

The ability of the selected jasmone-derived molecules to impair fungal growth was assessed on four fungal strains associated to esca disease in grapevine, previously characterized [25]. The investigated molecules were added to the Czapek Dox AgarCZA growth media at 25 and 50 µM concentrations. As reported in Figure 3, not all the molecules were effective against the fungal growth at 25 µM concentration: JTS and its Cu-complex (JTS-Cu) resulted in a complete impairment of colony development only in *P. chlamydospora*, almost completely inhibited by both the compounds up to 15 days. On the contrary, some variances were observed among the different species: 100% inhibition was achieved with JTS on *P. minimum* at 5 day post inoculation (dpi), but the effect was completely lost at 10 and 15 dpi. Conversely, JTS-Cu displayed a significant containment on fungal growth (about 30%) only at 10 and 15 dpi. The effect of JTS on *N. parvum* proved to be longer lasting effective, impairing the fungal growth from 65% (2 dpi) to 50% (7 dpi), whereas its complex JTS-Cu, although displaying a higher inhibition ability at 2 dpi (about 90%), completely lost its activity at 7 dpi. Both the cis-jasmone thiosemicarbazones slightly affect growth in *F. mediterranea* at 4 and 7 dpi, while at 2 dpi JTS-Cu was more effective (100% inhibition) than its ligand, showing that for this compound a growth recovery occurred.

Observations conducted on all the relevant species at 50 µM concentration of test compounds showed the achievement, by JTS and JTS-Cu, of a total impairment of colony development at every dpi of evaluation (Figure S1).

### 2.4. Antifungal Activity on Cereals Pathogens

**Figure 4:**
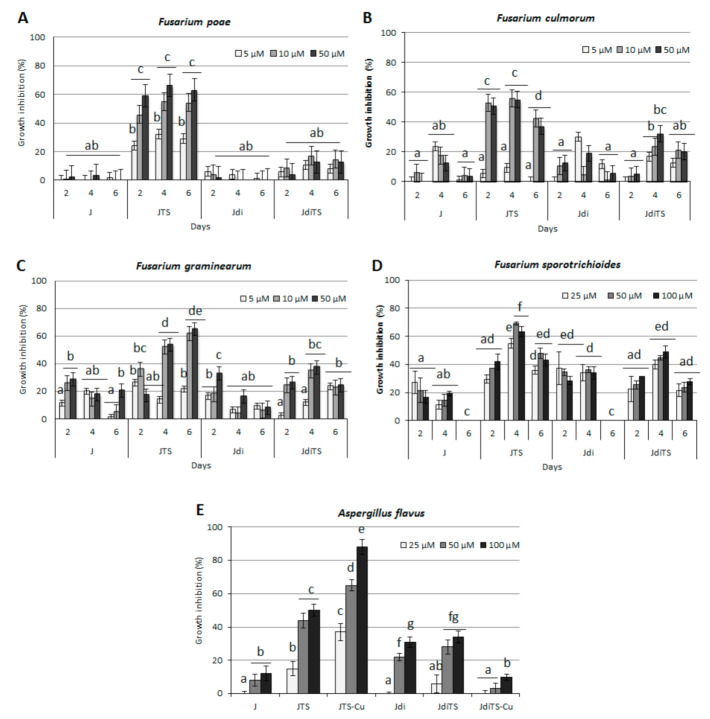
Effects of the compounds on the growth of cereals pathogenic fungi (**A**) *F. poae*, (**B**) *F. culmorum*, (**C**) *F. graminearum*, (**D**) *F. sporotrichioides* and (**E**) *A. flavus*. Culture medium was amended with an increasing concentration of molecules (from 5 to 100 µM) on the basis of the species; values are reported as inhibition percentage as compared to the control (DMSO amended cultures) ± S.D. Different letters indicate statistically significant differences (*p* < 0.05).

The ability of four selected molecules was tested against *Fusarium culmorum*, *F. graminearum, F. poae* and *F. sporotrichioides*, studying the effect on their growth in vitro. Four selected molecules (J, JTS, Jdi and JdiTS) were added to growth medium (PDA) at increasing concentrations (5, 25 and 50 µM); control plates contained DMSO at the relevant concentrations. Inhibition of fungal colony growth was monitored at 2, 4 and 6 days post inoculum. As given in Figure 4, compound JTS resulted to be the most effective in reducing fungal development: all strains of *Fusarium* spp. were strongly inhibited but the most affected was *F. poae*, being visibly reduced in growth from 2 dpi also at the lowest concentration (5 µM), while 25 and 50 µM concentrations reached respectively 45.9% and 59.1% inhibition and maintained a similar level until 6 dpi (54.2% and 63.0% respectively). Other compounds did not have any appreciable activity (Figure 4A). In *F. culmorum*, the fungistatic effect of JTS was obtained with 25 and 50 µM concentration and recorded on 2 and 4 dpi (52.6% and 55.6% respectively), while it slightly decreased at 6 dpi (Figure 4B); however, together with *F. graminearum*, this species resulted more susceptible than *F. poae* to other molecules: although below the threshold of 40% inhibition, J, Jdi and JdiTS exerted a time-dependent containment effect, that reached a maximum at 4 dpi (Figure 4C); J molecule inhibited the growth of *F. graminearum* in the initial growth period at 50 µM (28.7%) and of *F. culmorum* 4 dpi at 5 µM (23.4%). Jdi was most effective on *F. culmorum* at 5 µM, at 4 dpi (29.9%) and *F. graminearum* at 50 µM, at 2 dpi (33.1%). JdiTS mainly affected *F. graminearum* and *F. culmorum* growth on day 4 at 50 µM (37.5% and 32.0% respectively). Compound JTS resulted particularly effective in inhibiting *F. sporotrichioides* mycelium growth, reaching a maximum inhibition level of 70%, whereas J and Jdi reduced of 20%–30% the fungal growth only in the initial phase of the exposure. On the contrary, JdiTS showed an intermediate efficacy, reaching a maximum inhibition level of 50% after 4 days of exposure and only at the highest concentration (Figure 4D).

The whole panel of compounds was tested against *Aspergillus flavus*: when administrated at increasing concentration (from 25 to 100 µM) and evaluated for the inhibition of mycelium biomass production at 6 dpi, neither J nor Jdi molecule showed any fungistatic effect, while the relevant ligands proved to affect fungal growth at a higher level (Figure 4E). Complexation with copper induced an increase of the fungistatic effect only in the case of JTS-Cu, while almost reducing JdiTS fungistatic activity.

### 2.5. Antifungal Activity on Horticultural Crop Pathogens—Sclerotinia spp., V. dahliae, Rhizoctonia solani and F. oxysporum

**Figure 5:**
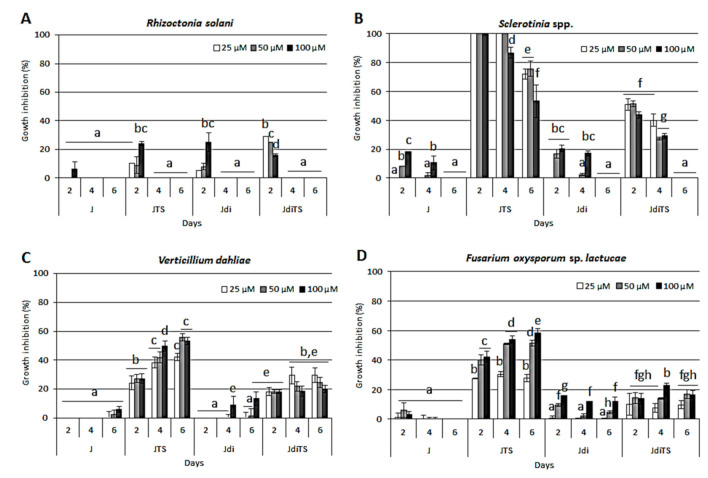
Effect of compounds on the growth of horticultural crop pathogens (**A**) *R. solani*, (**B**) *Sclerotinia* spp., (**C**) *V. dahliae* and (**D**) *F. oxysporum*. Culture medium was amended with increasing concentrations of molecules (from 25 to 100 µM) on the basis of the species; values are reported as inhibition percentage as compared to the control (DMSO amended cultures) ± S.D. Different letters indicate statistically significant differences (*p* < 0.05).

The antimicrobial potentialities of J, JTS, Jdi and JdiTS at 25, 50 and 100 µM concentration were evaluated, in terms of colony growth inhibition, on four fungal species affecting a wide range of horticultural crops; Figure 5 reports the percentages of growth inhibition respect to control, obtained at 2, 4 and 6 dpi. As major points, it was found that all the fungal species showed a similar trend of susceptibility to all compounds, even at different extent: *Rhizoctonia solani*, the less sensitive, touched the maximum level of growth inhibition (20–30%) when exposed to JTS, Jdi and JdiTS. On the contrary, *Sclerotinia* proved to be the most affected by the exposure to compounds. Additionally, not all the molecules were effective in containing the fungal growth: in fact, only JTS resulted in a complete impairment of colony development for *Sclerotinia* and *V. dahliae*.

### 2.6. Interference with Fungal Secondary Metabolism—Effect on Mycotoxin Production and Sclerotia Development

**Figure 6:**
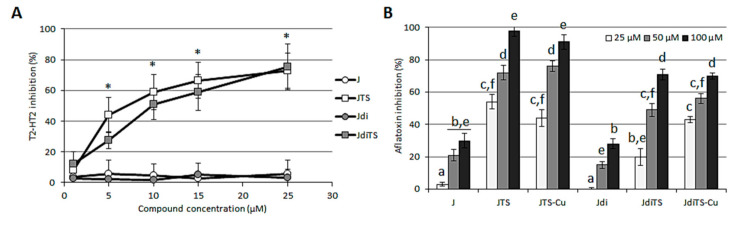
Effect of compounds on the mycotoxin production in two cereals pathogenic fungi. Toxins T-2 and HT-2 were quantified in *F. sporotrichioides* cultures exposed to increasing concentration (from 1 to 25 µM) of J, JTS, Jdi and JdiTS compounds (**A**). Aflatoxins were quantified in *A. flavus* cultures exposed to increasing concentration (from 25 to 100 µM) of all the compounds (**B**). Values are reported as inhibition percentage as compared to the control (DMSO amended cultures) ± S.D. Asterisks or different letters indicate statistically significant differences (*p* < 0.05).

Besides the inhibitory potential against mycelium growth, the possible interference of some compounds with fungal secondary metabolisms was assessed: more specifically, the possible impairment of mycotoxins biosynthesis and sclerotia production in vitro was evaluated for a few relevant species. The production of T-2/HT-2 toxin by *F. sporotrichioides* treated with different concentrations of J, Jdi, JTS and JdiTS showed to be differently affected: the exposure to natural ketones did not induced any reduction, even at the highest concentrations (Figure 6A). On the contrary, both ligands were effective in lowering the mycotoxin biosynthesis: from the 40% inhibition at 5 µM to 75% at 25 µM concentration. *Aspergillus flavus* response to molecules provided interesting findings about ligands and their metal complexes: as observed in *F. sporotrichioides*, ketones reached a maximum of 30% inhibition, at 100 µM, while a stronger containment of aflatoxin (up to 98%) was achieved with JTS and JdiTS. However, the addition of a copper ion to the thiosemicarbazonic molecule did not enhance their anti-mycotoxigenic activity (Figure 6B). Coherently with aflatoxin inhibition, when tested on sclerotia biogenesis J and Jdi resulted completely ineffective and both ligands proved to be able to reduce sclerotia production up to 71.9% and 62.5% (for JTS and JdiTS respectively); surprisingly, contrariwise to what observed for the aflatoxin metabolism, exposure to JTS-Cu determined an enhanced activity with respect to JTS, while the copper complex JdiTS-Cu resulted in a complete abatement of JdiTS effect (Table 3).

When evaluated on *Sclerotinia* spp., the two ligands showed a different behavior with respect to *A. flavus*: the inhibitory activity on the sclerotia development was similar for JTS and JdiTS (Table 3) and determined a reduction of the number of sclerotia *per* colony area (cm^2^) of 55.3% and 52.6% respectively.

## 3. Discussion

Jasmonic acid (JA) and its derivatives (such as methyl-jasmonate) belong to the plant oxylipins family, a large group of metabolites derived from polyunsaturated fatty acids commonly associated with the modulation of defense response [26]. Recently, the role of oxylipins in plant defense against biotic stressors has been rediscovered, suggesting, in addition to the induction and regulation of gene expression in the plant host, a direct antimicrobial effect on microbial pathogens. On this path, in vitro investigations on growth inhibitory activity of various natural oxylipins proved that some of them are able to heavily affect mycelial growth and spore germination in a number of eukaryotic phytopathogens, indicating a contribution of such compounds to host resistance through related but different mechanisms [27]. Six molecules structurally related to JA was thus tested against a set of 13 phytopathogenic fungal species, with the aim of compare the potential of modified JA derivatives in controlling fungal diseases of economically important crops. Due to their synthesis low yield, the two metal complexes were tested only on pathogens relevant to maize and grape, since they are considered model systems for herbaceous/monocots and woody/dicots respectively. Relative to the biological activity of compounds on both primary (fungal growth) and secondary metabolisms (mycotoxins accumulation and sclerotia biogenesis), some interesting differences between the JA-derivatives were found—natural ketones did not show any fungistatic effect on fungal pathogens affecting grapevine and linked to grapevine esca disease, as well as pathogens affecting horticulture crops; however, a slight inhibitory activity was recorded on cereals contaminating species (*Fusarium* and *Aspergillus* spp). Thiosemicarbazones were found more effective than their parent compounds, even if to a different, species-specific extent; JTS was undoubtedly the most promising, being able to prejudice the growth, at the highest level, of almost all the pathogens tested, with the only exception of *R. solani*, which, indeed, revealed to be the less susceptible to the exogenous administration of JA derivatives.

From a developmental perspective, the results obtained analyzing the response of *A. flavus* to our compounds were intriguing: in fact, if in *F. sporotrichioides* the inhibitory effect on toxin T2-HT2 accumulation seemed to be coupled with a comparable fungistatic activity, in *A. flavus* the highest fungistatic and anti-aflatoxigenic activity of thiosemicarbazonic ligand—in comparison with its natural ketone—was observed for JTS but not for JdiTS, that, with respect to Jdi, showed an increased effect on aflatoxin accumulation but not on fungal growth suppression. Even more interesting was the behavior of the two copper complexes that, in contrast with their free ligands, showed a different activity trend—the complexation with the metallic nucleus appeared in fact to determine an intensification of the fungistatic potential only for JTS-Cu, whose inhibitory effect on aflatoxin accumulation seems to be justified by the mycelial reduction. On the other hand, JdiTS did not differ from JdiTS for the anti-aflatoxigenic activity but the addition of the copper ion almost deleted its fungistatic effect. This antagonist effect of JA-derivatives against aflatoxins accumulation in *A. flavus* with no apparent effect on mycelial growth was already described [28].

Results obtained for the esca-related pathogens are of great interest: esca syndrome is now becoming the most devastating issue in viticulture, with an impact on both yield and quality [29], since the nature of this syndrome, which derives from the simultaneous colonization of the woody tissues from several fungi, makes it a difficult target for common antimicrobial treatments [30]. For this reason, up to date, a cure for this syndrome is not available and only preventive techniques can be undertaken. In this light, the ability of JAs to impair fungal growth can be implemented and exploited to counteract the esca syndrome.

Interesting results in terms of inhibitory effect were also obtained against *Fusarium* spp., the cause of the economically important disease of cereals (FHB) and mycotoxins contaminant agent: the high fungistatic activity of JTS against *Fusarium* spp. strains, which was maintained even after 6 days of growth, suggests that such compound can be used in the future to formulate new fungicides for the protection of cereal ears against FHB; additionally, the observation of the thiosemicarbazones different effectiveness depending on fungus species confirmed the findings previously described [31,32].

Intriguing observations can also be raised about the antioxidant activity if compared to sclerotia biogenesis impairment, since in many fungal species the redox balance is considered a prominent factor involved in the control of secondary metabolism and several synthetic and natural compounds that modulate secondary pathways—such as AF biosynthesis and sclerotia biogenesis—proved to possess a scavenging activity against ROS. In particular, the sclerotia metamorphosis in filamentous fungi appears to be triggered by oxidative stress [33,34,35]. In this study, no correlation was found between the predicted antioxidant scavenging ability of compounds, as determined in vitro with the DPPH assay and their effect on the secondary metabolism (intended as both sclerotia biogenesis and mycotoxin biosynthesis), consistently with previously obtained results [23,36]: the Cu ion provided the thiosemicarbazones with a scavenging potential otherwise absent in both ketones and ligands. Copper was thought to be the redox active center of compounds but actually it resulted in a highly variable in vivo effect. In consideration of these findings, the hypothesis of a correspondence between in vitro scavenging potential of Jasmone-derived TSs and their in vivo biological effect on *A. flavus* has to be argued: hereto, the anti-aflatoxigenic effect of JTS and JdiTS is accompanied by the inhibition of sclerotia biogenesis, suggesting that their target(s) could be involved in biochemical processes shared by both the aflatoxin and the sclerotia metabolism. Conversely, the highly significant anti-toxigenic effect of JTS-Cu seems to be unspecific and mainly due to an impairment of fungal primary metabolism, that in turn strongly affects aflatoxin biosynthesis and sclerotia production, as a consequence. In this sense, JdiTS-Cu resulted in the most interesting compound—the outcomes suggest a specificity of action whose target, differently from JTS and JdiTS, might be located in a metabolic knot closely related to the aflatoxin biosynthetic pathway, as supported by the observation that the prominent containment activity of this compound on aflatoxin accumulation is not supported by a significant impact on sclerotia development and hyphae growth.

## 4. Materials and Methods

### 4.1. Chemical Synthesis

The desired TSs (ligands) were obtained by mixing an equimolar amount of thiosemicarbazide with the appropriate aldehyde in absolute ethanol. A small amount of hydrochloric acid was added to catalyze the condensation. The mixture was refluxed under stirring for 8 h and left overnight at 0 °C. The precipitate was filtered out, washed with cold ethanol and dried under vacuum.

*Cis-jasmonethiosemicarbazone*: thiosemicarbazide (0.22 g, 2.4 mmol), cis-jasmone (0.40 mg, 2.4 mmol). White powder. Yield 26%. Mp. 149 °C. Fourier-transform infrared spectra (FT-IR) (cm^−1^): 3401 (s), 3130 (m), 2964 (m), 1590 (s), 1507 (s) 874 (m). ^1^H-NMR (δ, ppm; DMSO-d_6_): 0.93 (t, J = 7.5 Hz, 3H), 1.89 (s, 3H), 2.13 (m, 2H), 2.44 (m, 2H), 2.59 (m, 2H), 2.98 (d, J = 9.2 Hz, 2H), 5.28 (m, 2H), 734 (s, 1H), 8.02 (s, 2H), 9.87 (s, 1H).

*Dihydrojasmonethiosemicarbazone*: thiosemicarbazide (0.22 g, 2.41 mmol), dihydrojasmone (0.40 g, 2.41 mmol). Pale yellow powder. Yield 41%. Mp. 177 °C. FT-IR (cm^−1^): 3403 (s), 3130 (m), 2925 (m), 2852 (s), 1590 (m), 1507 (s), 718 (m). ^1^H-NMR (δ, ppm; DMSO-d_6_): 0.85 (t, J = 7.0 Hz, 3H), 1.25 (m, 4H), 1.37 (m, 2H), 2.22 (t, J = 7.4 Hz, 2H), 2.43 (m, 2H), 2.58 (m, 2H), 7.30 (s, 1H), 8.01 (s, 2H), 9.83 (s, 2H).

Metal complexes were obtained by dripping the solution containing the copper salt into the ligand solution, which rapidly turned to dark brown. The mixture was stirred at room temperature for 2 h and then the solvent was removed under reduced pressure. The solid formed was collected and washed with diethyl ether, then dried under vacuum.

*Bis(cis-jasmonethiosemicarbazonate) Cu(II)*: copper(II) acetate (0.04 g, 0.21 mmol), 38 (0.10 g, 0.42 mmol). Dark brown powder. Yield 95%. FT-IR (cm^−1^): 3416 (s), 2959 (m), 1570 (m), 1511 (m), 701 (m). ESI-MS (+) m/z calc. 537.28, found 537.39. *Bis(dihydrojasmonethiosemicarbazonate) Cu(II)*: copper(II) acetate (0.04 g, 0.21 mmol), 39 (0.10 mg, 0.42 mmol). Dark brown powder. Yield 74%. FT-IR (cm^−1^): 2920 (s), 2851 (m), 1550 (m), 618 cm^−1^ (m). ESI-MS (+) m/z calc. 541.31, found 542.42.

All compounds were dissolved in di-methyl sulfoxide (DMSO) to achieve 10 mM stocks.

### 4.2. X-ray Analysis

X-ray diffraction data collection of compound JTS was carried out with a Bruker-Siemens SMART AXS 1000 diffractometer equipped with a charged-coupled device CCD detector, Mo Kα radiation (λ = 0.71069). The phase problem was solved by direct methods and the structure was refined by full-matrix least-squares on all F2 using SHELXL97 [37] as implemented in the Olex package [38]. Figures have been obtained using the Mercury software [39].

Crystal data: triclinic, *P*_ī_, a = 8.164(5) Å, b = 15.645(9) Å, c = 16.434(9) Å, α = 84.723(10), β = 82.036(10), γ = 84.632(10), V = 2063(2) Å^3^; Z =2; dcalc = 1.146 mg/cm^3^, F(000) = 768, mu = 2.15, Tot. refl. = 19,159, hkl range = −9 < h < 9, −19 < k < 19, −20 < l < 20; Theta range 1.25–25.80, unique reflections = 7881, number of parameters = 433, GooF = 1.033, R = 0.0835, wR2 = 0.2039.

CCDC 2,019,086 contains the supplementary crystallographic data for this compound. These data can be obtained free of charge from The Cambridge Crystallographic Data Centre via www.ccdc.cam.ac.uk/data_request/cif.

### 4.3. Scavenging Activity Assay (DPPH)

A 90 μM methanol solution of 2,2-diphenyl-1-picrylhydrazyl radical (DPPH) was prepared: an aliquot of 2 mL was then incubated at room temperature with 12.5 μL of DMSO-dissolved sock solution of each compound corresponding to 20, 30, 40 and 50 μM concentration. The scavenging activity of the compound against the DPPH was observed with the decrease in absorbance measured at 518 nm. A solution of ascorbic acid at 0.3 mM was used as positive control to determine the maximal decrease in DPPH absorbance. Values were expressed in percentage of inhibition of DPPH absorbance in relation to the control values without the compound (ascorbic acid maximal inhibition was considered 100% of inhibition).

### 4.4. Biological Assays

#### 4.4.1. Microorganisms

*Phaeoacremonium minimum*, *Neofusicoccum parvum*, *Phaeomoniella chlamydospora* and *Fomitiporia mediterranea* were chosen amongst grapewine diseases-associated fungal species which were previously characterized among the Research Centre for Viticulture and Enology (CREA-VE, Conegliano Veneto (TV), Italy) esca-associated fungal collection [25]. *Fusarium poae* (OZ45), *F. culmorum* (OZ47), *F. graminearum* (OZ161) strains were isolated from barley and oat grains and deposited in the fungal collection of Plant Protection Department at the University of Life Sciences in Lublin (Poland).

*Aspergillus flavus* strains CR10+ and TOφ strains, previously characterized and described [40,41], belong to the fungal collection of the Laboratory of Mycotoxicology (Dept. of Chemistry, Life Sciences and Environmental Sustainability, University of Parma, Italy). Strains of *Fusarium sporotrichioides*, *Rhizoctonia solani*, *Fusarium oxysporum* f.sp *lactucae*, *Verticillium dahliae* and *Sclerotinia* spp. were provided by the Università Cattolica del Sacro Cuore (Piacenza, Italy). All fungal strains were maintained on Potato Dextrose Agar (Difco, Becton, Dickinson & Co., Le Pont-de-Claix, France).

#### 4.4.2. Grapewine Diseases-Associated Fungal Species Growth Inhibition Assay

Fungal isolates were grown on CYA media as previously reported [42]. Compounds were added to the CYA growth media at 25 and 50 µM concentrations, then fungal growth was compared to a mock amended plate (control). Fungi were grown for at least 3 subcultures prior to be used in the inhibition assay. For *P. minimum* and *P. chlamydospora* conidia were harvested on a 15 days old colony using water with 0.02% Tween 20 and 0.05% agar. The suspension was then then diluted to 10^6^ conidia per ml and 2 µL were used to inoculate plates. Since *N. parvum* and *F. mediterranea* do not produce conidia the inoculum was made by mycelia plugs of 25 mm diameter harvested from the edge of 5 days old colonies. Due to different growth rates, *P. minimum* and *P. chlamydospora* were checked at 5, 10 and 15 days after inoculation whereas for *N. parvum* and *F. mediterranea* growth measurements were done at 2, 4 and 7 days post inoculation. Results were expressed as percentage of growth inhibition compared to the control. Experiments were performed in triplicate.

#### 4.4.3. *Fusarium* spp. and Soil-Borne Pathogens Growth Inhibition Assay

The mycelial growth of *Fusarium* strains, *Rhizoctonia solani*, *Verticillium dahliae* and *Sclerotinia* spp. was evaluated on Potato Dextrose Agar (PDA) medium amended with the compounds at increasing concentration (from 5 to 100 µM). The Petri dish method was applied, according to Thanassoulopoulos et al. [43]. Control cultures were prepared amending PDA with equivalent volumes of DMSO (≥99.9%, Sigma Aldrich, St Louis, MO, USA), ranging from 0.25% to 1%. The experiments were conducted twice in triplicate in 60 mm petri dishes inoculated in the center with 8 mm PDA plugs from actively growing cultures. Each molecule was added in appropriate concentration to sterile Petri dishes of 90 mm diameter poured with liquid medium and then inoculated with fungi colonies of 3 mm diameter. Inoculum originated from 10-day-old single-spore colonies of tested species grown on PDA (Difco, Becton, Dickinson & Co., France). Three replications of each experimental combination were made. Plates were incubated up to 10 days at 25 °C, measuring colonies at 2, 4 and 6 days post-inoculation. The antifungal effect was expressed as percentage inhibition, calculated according to the formula: I = [100 − (T/C)] × 100, where I is the percentage of inhibition, C is the control plate colony diameter in mm and T is the treated plate colony diameter in mm [44].

#### 4.4.4. *F. sporotrichioides* T2-HT2 Toxins Determination

The whole mycelium, including the underlying PDA, was collected from control (PDA amended with DMSO ranging from 0.01% to 1%) and treated plates (PDA amended with J, Jdi, JTS and JdiTS ranging from 1 to 100 µM) six days after inoculation with *F. sporotrichioides*. The collected samples were extracted with 5 volumes of 70% methanol and vigorously shaken for 15 min before filtering. The amount of T-2, HT-2 toxins (as sum of toxins) was determined using the kit Veratox^®^ for T-2, HT-2 (Product code 8230, Neogen Corporation, Lansing, MI, USA) a competitive direct enzyme-linked immunosorbent assay (ELISA). The photometric reading was done at 630 nm, according to the manufacturer’s instructions.

#### 4.4.5. Aflatoxin, Biomass and Sclerotia Inhibition Assays in *A. flavus*

For biological assays on *Aspergillus flavus* the aflatoxigenic strain CR10 and the non-aflatoxigenic strain TOφ were used [42,43]. Aflatoxin production was evaluated directly in the culture medium (coconut clarified medium, CCM) by a microplate high throughput procedure [45]. Briefly, increasing concentrations of compounds were added to CCM-filled microplate wells, then CCM cultures were incubated at 25 °C in the dark, under stationary conditions for 6 days. AFs accumulation was determined by fluorescence emission (TECAN SpectraFluor Plus microplate reader, Männedorf, Switzerland; λ_ex_ = 360 nm; λ_em_ = 465 nm; manual gain = 83; lag time = 0 μs; number of flashes = 3; integration time = 200 μs). Compounds were tested at 25, 50 and 100 µM concentration. Results were expressed as percentage inhibition respect to control (0.25, 0.5 and 1% *v/v* DMSO-treated cultures respectively). Cultures were inoculated in quadruplicate and experiments were conducted in triplicate.

Biomass production was assessed by recovering, after six days of incubation, single mycelia from the microplate wells used for aflatoxin accumulation—samples were slightly dried on hands paper and weighted and then values were converted in percentage inhibition respect to control (DMSO-treated cultures). Cultures were inoculated in quadruplicate and experiments were conducted in triplicate.

#### 4.4.6. Sclerotia Inhibition Assays in *A. flavus* and *Sclerotinia* spp.

Sclerotia biogenesis in *A. flavus* was measured by point-inoculating 5 μL of the aflatoxigenic strain CR10 conidial suspension (approximately 10^6^ conidia/mL) in Petri dishes (Ø = 5 cm) poured with Czapek Dox Agar (CZA) medium added with 100 μM compounds; control plates were amended with 1% (v/v) DMSO. After two weeks of incubation at 30 ˚C in darkness, sclerotia were manually scraped from the colonies surface, washed with a 70% ethanol solution, dried for three days at 60 °C then weighted. Inhibition rate on sclerotia production was expressed as percentage respect to the control (mg/colony area). Plates were inoculated in triplicate. For *Sclerotinia* spp., a mycelium plug was cultured in Petri dishes (Ø = 5 cm) poured with Czapek Dox Agar (CZA) medium added with 100 μM compounds and incubated as above. Sclerotia were manually collected from plates and counted. Inhibition rate was expressed as a percentage with respect to the control (nr/colony area).

#### 4.4.7. Statistical Analysis of Biological Data

One-way analysis of variance (Past 3.x software) was used for data analyses. Tukey’s test was applied to the data relative to mycelial growth, mycotoxin accumulation and sclerotia production; differences were regarded as significant at *p* < 0.05.

## 5. Conclusions

Recently, in Europe even more restriction rules have been introduced for Cu containing molecules in the management of defense strategies against fungal pathogens. This because Cu, if massively used, tends to accumulate in soils, so far becoming a pollutant for the environment threatening the human health [46]. For these reasons, an urgent need for the development of alternatives to reduce chemical inputs and environmental Cu accumulation is required, particularly in agricultural soils [47]. In the last decades, research efforts led to a better understanding of molecular and biochemical mechanisms driving plant and fungal response to certain phytohormones, including JA; however, only a few findings suggested a possible direct interaction between JA-related compounds and fungal plant pathogens, also in terms of mycotoxins production. Our results definitely assessed how some thiosemicarbazonic JA-derived molecules (and in particular JTS) can exert a containment effect on both fungal growth/development and secondary metabolism in phytopathogenic species infecting agriculturally important crops, such as grape, cereals and horticultural species, being thus extremely interesting for plant health control strategies and food security purposes.

## Figures and Tables

**Table 1:** The different hydrophobic chain dihedral angles for the three independent molecules.

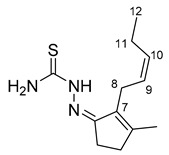		**A**	**B**	**C**
	C7–C8–C9–C10	138.9 (8)°	−125.1 (8)°	−130.9 (6)°
	C8–C9–C10–C11	−4.3 (16)°	−2.0 (15)°	−2.0 (15)°
	C9–C10–C11–C12	−153.6 (13)°	134.3 (11)°	123.9 (8)°

**Table 2:** Average bond lengths in the thiosemicarbazone moiety of the three molecules.

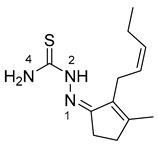	**Atoms**	**Average Length (Å)**
	C-N4	1.320
	C-S	1.695
	C-N2	1.346
	N2-N1	1.389

**Table 3:** Effect of compounds on sclerotia biogenesis. Sclerotia biomass (*A. flavus*) or number (*Sclerotinia* spp.) was determined in cultures exposed to 100 µM concentration of compounds. Values are reported as inhibition percentage as compared to the control (DMSO amended cultures) ± S.D. Asterisks indicate statistically significant differences (*p* < 0.05).

	*Aspergillus flavus*	*Sclerotinia* spp.
Compound	Inhibition (%) mg/Colony Area (cm^2^)	Inhibition (%) nr/Colony Area (cm^2^)
JTS	71.9 ± 3.0 *	55.3 ± 7.1 *
JTS-Cu	96.9 ± 1.6 *	n.d.
JdiTS	62.5 ± 5.7 *	52.6 ± 3.5 *
JdiTS-Cu	0.0 ± 0.2	n.d.

## Supplementary Materials

Supplementary materials can be found at https://www.mdpi.com/1422-0067/21/22/8681/s1, Figure S1: Effect of compounds on the growth of esca disease-linked species. Culture medium was amended with molecules at 50 μM concentration; values are reported as inhibition percentage as compared to the control (DMSO 0.5% *v/v* amended cultures) ± S.D. Different letters indicate statistically significant differences (*p* < 0.05).
