# Supplementary figures and images for: Double Gamers—Can Modified Natural Regulators of Higher Plants Act as Antagonists against Phytopathogens? The Case of Jasmonic Acid Derivatives
Source: Int J Mol Sci. 2020 Nov 17;21(22):8681. doi: 10.3390/ijms21228681 (PMC7698523; doi:10.3390/ijms21228681)

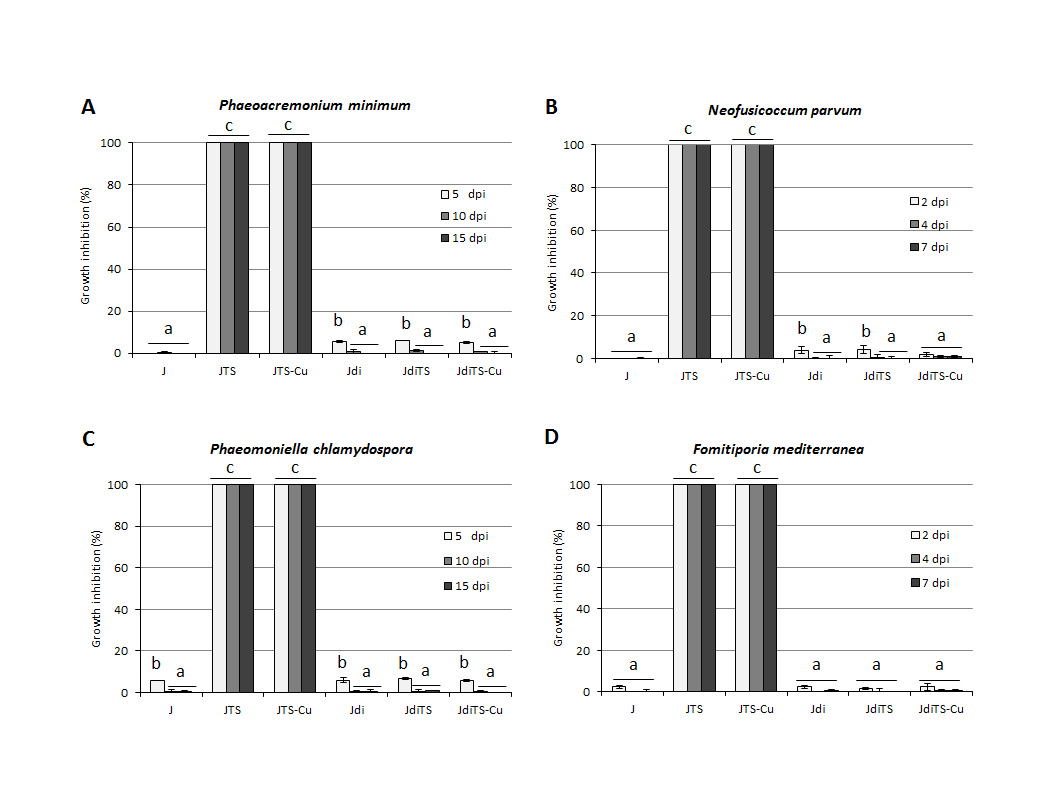

Supplement: Supplementary file 1 [file ijms-21-08681-s001.zip › ijms-982378-supplementary.jpg]
